# Newspaper Law Decisions

**Published:** 1877-11

**Authors:** 


### Newspaper Law Decisions.

1. Any person who takes a paper regularly from the post-office—whether directed to his name or another's or whether he has subscribed or not—is responsible for the payment.

2. If a person orders his paper discontinued, he must pay all arrearages, or the publisher may continue to send it until payment is made, and collect the whole amount, whether the paper is taken from the office or not.

3. The courts have decided that refusing to take newspapers or periodicals from the post-office, or removing and leaving them uncalled for, is *prima facia* evidence of intentional fraud.

:o:

We have received from the publishers, H. C. Lea, and Lindsay & Blakiston, a number of new medical works, through T. H. Butler, whose new store is located at 307 Main street, where may be found all the latest standard medical publications.

